# Chromosome-Level Genome Assembly of the Blue Mussel *Mytilus chilensis* Reveals Molecular Signatures Facing the Marine Environment

**DOI:** 10.3390/genes14040876

**Published:** 2023-04-07

**Authors:** Cristian Gallardo-Escárate, Valentina Valenzuela-Muñoz, Gustavo Nuñez-Acuña, Diego Valenzuela-Miranda, Fabian J. Tapia, Marco Yévenes, Gonzalo Gajardo, Jorge E. Toro, Pablo A. Oyarzún, Gloria Arriagada, Beatriz Novoa, Antonio Figueras, Steven Roberts, Marco Gerdol

**Affiliations:** 1 Center for Aquaculture Research, University of Concepción, Concepción 4070386, Chile; 2 Laboratorio de Genética, Acuicultura & Biodiversidad, Departamento de Ciencias Biológicas y Biodiversidad, Universidad de Los Lagos, Osorno 5310230, Chile; 3 Facultad de Ciencias, Instituto de Ciencias Marinas y Limnológicas (ICML), Universidad Austral de Chile, Valdivia 5110566, Chile; 4 Centro de Investigación Marina Quintay (CIMARQ), Universidad Andres Bello, Quintay 2340000, Chile; 5 Instituto de Ciencias Biomédicas, Facultad de Medicina, Universidad Andrés Bello, Santiago 8370186, Chile; 6 FONDAP Center for Genome Regulation, Santiago 8370415, Chile; 7 Instituto de Investigaciones Marinas (IIM), Consejo Superior de Investigaciones Científicas (CSIC), 36208 Vigo, Spain; 8 School of Aquatic and Fishery Sciences (SAFS), University of Washington, Seattle, WA 98195, USA; 9 Department of Life Sciences, University of Trieste, 34127 Trieste, Italy

**Keywords:** chromosome-level assembly, comparative genomics, transcriptomics, *Mytilus chilensis*

## Abstract

The blue mussel *Mytilus chilensis* is an endemic and key socioeconomic species inhabiting the southern coast of Chile. This bivalve species supports a booming aquaculture industry, which entirely relies on artificially collected seeds from natural beds that are translocated to diverse physical–chemical ocean farming conditions. Furthermore, mussel production is threatened by a broad range of microorganisms, pollution, and environmental stressors that eventually impact its survival and growth. Herein, understanding the genomic basis of the local adaption is pivotal to developing sustainable shellfish aquaculture. We present a high-quality reference genome of *M. chilensis*, which is the first chromosome-level genome for a Mytilidae member in South America. The assembled genome size was 1.93 Gb, with a contig N50 of 134 Mb. Through Hi-C proximity ligation, 11,868 contigs were clustered, ordered, and assembled into 14 chromosomes in congruence with the karyological evidence. The *M. chilensis* genome comprises 34,530 genes and 4795 non-coding RNAs. A total of 57% of the genome contains repetitive sequences with predominancy of LTR-retrotransposons and unknown elements. Comparative genome analysis of *M. chilensis* and *M. coruscus* was conducted, revealing genic rearrangements distributed into the whole genome. Notably, transposable Steamer-like elements associated with horizontal transmissible cancer were explored in reference genomes, suggesting putative relationships at the chromosome level in Bivalvia. Genome expression analysis was also conducted, showing putative genomic differences between two ecologically different mussel populations. The evidence suggests that local genome adaptation and physiological plasticity can be analyzed to develop sustainable mussel production. The genome of *M. chilensis* provides pivotal molecular knowledge for the *Mytilus* complex.

## 1. Introduction

The blue mussel *M. chilensis* (Hupé, 1854) is Chile’s endemic, ecological, and socioeconomic key species that leads the national shellfish aquaculture. The farming of *M. chilensis* involves the use of ropes or nets that are suspended from rafts or longlines in the sea. The mussels attach themselves to the ropes or nets and grow there until they reach market size, which typically takes about 18–24 months. The mussels feed on natural phytoplankton in the water, and their growth and survival depend on the availability and quality of this food source [1]. In Chile, the aquaculture of *M. chilensis* is an important industry, with production levels that have been steadily increasing over the years. The mussels are exported to many countries around the world, and they are highly valued for their flavor and nutritional value. The aquaculture mussel production in Chile was about 400,000 tons in 2020 [2].

However, the success of mussel aquaculture production in Chile is threatened by a wide range of microorganisms [3,4,5], marine pollution [6], and climate variability that can impact the larval settlement and growth of mussel populations [7,8,9]. To cope with those stressors, mussels and marine invertebrates produce two-component responses, a specific response to the stressor and a more general response involving immune and endocrine pathways [10]. Multi-stressors’ impacts have been predicted to have additive, synergetic, or antagonistic effects on marine organisms’ physiology [11]. These different effects are directly linked to the amount of time between the occurrence of stressors, their intensity, and the organism’s ability to return to homeostasis before a new stressor occurs [12]. Despite these predictions, meta-data analyses show that most of the studied multi-stressors had synergetic effects on organisms’ physiology. Notably, isolated effects of environmental stressors or pathogen infection on the mussel immune system have been extensively studied [13,14,15,16,17,18,19,20]. However, mussels’ immune response to the combination of stressors remains unexplored. The interplaying between the immune system and multi-environmental stressors such as ocean acidification, hypoxia, marine heatwaves, harmful algal blooms, and pathogen infections requires physiological, cellular, and molecular tools that uncover the complexity of mussel biology. High-quality genome assembly at the chromosome level is pivotal to driving the scientific community in this endeavor and mussels represent an outstanding model species. For instance, mussel species display morphologically conserved karyotypes, and recent studies have evidenced whole-genome duplication events [21,22]. Compared with other bivalves, the reported mussel genomes share relatively large genome sizes characterized by high heterozygosity and expanded mobile elements [23,24,25,26,27]. Unfortunately, these genome features challenge the assembly efforts to avoid genome fragmentation. Up to now, chromosome-level genome assembly for Mytilidae has only been reported for the congeneric species *M. coruscus* [28] and the zebra mussel *Dreissena polymorpha* [27]; meanwhile, other members of the family have been reported as highly contiguous reference assemblies at contig level [29,30,31]. Interestingly, the presence–absence variation (PAV) phenomenon has recently been reported for *M. galloprovincialis*, where a pan-genome composed of 20,000 protein-coding genes was observed in conjunction with dispensable genes that are entirely missing in some mussels [32].

The Chilean blue mussel *M. chilensis*, a close relative of the *M. edulis* species complex of the northern hemisphere [33,34], represents an iconic species to explore key questions in ecology [35], ecophysiology [36] and adaptative genomics [37,38]. It is a keystone taxon in the ecosystem regulating phytoplankton and nutrient flow and contributes to remineralizing organic deposits in the sediment [39,40]. It inhabits rocky substrates in the intertidal and subtidal zones along the southern Pacific Ocean from latitude 38S to 53S [41]. As a gonochoric species with an annual gametogenic cycle, sexual maturity occurs in spring–summer, where planktonic larvae can drift between 20 and 45 days before settlement [42]. Dispersal potential has been estimated to be up to 30 km, allowing different degrees of gene flow among mussel populations [43].

In this study, PacBio sequencing, and Hi-C scaffolding technology were jointly used to assemble the first chromosome-level reference genome of *M. chilensis*. Moreover, we conducted a comparative genomics study among reported genome mussel species and analyzed the molecular signatures in two mussel populations facing distinct physical–chemical ocean conditions. Genomic features revealed putative chromosome rearrangements among mussel species, suggesting phylogenetic relationships for retrotransposons in Mytilidae. Specifically, Steamer-like elements were classified as LTR-retrotransposons because they contain long terminal repeats (LTRs) at both ends of their DNA sequence. The chromosome-level genome assembly of *M. chilensis* is a useful resource for genome-wide association studies (GWAS) as a powerful tool used in genetics to identify genetic variants associated with complex traits or diseases. Notably, this study revealed the most differentially expressed genes and single nucleotide polymorphisms found in *M. chilensis* populations, revealing specific transcriptome profiles associated with metabolism and immune-related genes. The knowledge gained in this research will provide pivotal information for exploring how the marine environment drives phenotypic plasticity, which can be associated with genome adaptation in mussel populations.

## 2. Materials and Methods

### 2.1. Sample Collection, NGS Libraries, and Sequencing

**Figure 1:**
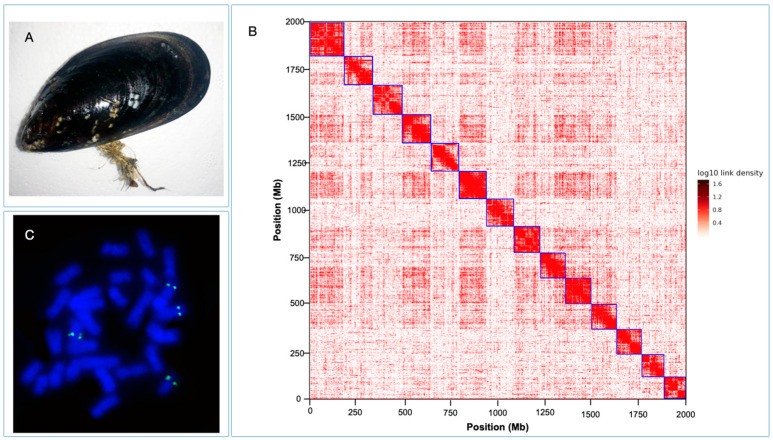
(**A**) Photograph of the native blue mussel *Mytilus chilensis*. (**B**) Metaphasic chromosomes from mussel larvae samples and mapping of 28S-rDNA by fluorescence in situ hybridization. (**C**) Heatmap of chromosome interaction intensity in the blue mussel Hi-C assembly. The *x*- and *y*-axis represent the length of the chromosomes. The color bar represents the Hi-C contact density.

Adult *M. chilensis* were collected from a natural bed in Puerto Mont (41°48′ S–73°5′ W), Chile (Figure 1A). Five mussels were selected for whole-genome sequencing using 1 mL of hemolymph collected from each specimen to reduce the heterozygosity. The samples were centrifuged at 1200 RPM to isolate the hemocyte cells and preserved by liquid nitrogen. High-quality DNA was isolated using the Qiagen DNA purification kit (QIAGEN, Germantown, MD, USA) following the manufacturer’s instructions and quantified by a Tape Station 2200 instrument (Agilent, Santa Clara, CA, USA). DNA samples >9.5 in DNA integrity numbers (DIN) were selected for library preparation. Furthermore, 50 individuals per population were sampled from Cochamó (41°28′ S–72°18′ W) and Yaldad (43°07′ S–73°44′ W), in southern Chile, to isolate RNA and explore molecular signatures associated with the local genome adaptation. Herein, these mussel populations inhabit contrasting oceanic environments characterized by an estuary with continuous input of freshwater and vertical stratification, and a bay exposed to open sea influence, respectively. The temporal and spatial variability of sea surface temperature (SST) around Puerto Montt, Chiloé island, and at the Yaldad and Cochamó sites were analyzed using satellite images. Data on sea surface temperature in the region of interest were obtained from MUR-SST (Multi-scale Ultra-High-Resolution SST) distributed by NOAA through its ERDDAP platform. The MUR-SST images have a spatial resolution of 1 km and a temporal resolution of 1 day. In situ temperature (°C) and salinity (PSU) seawater measurements were obtained for both locations between 2018 and 2019. The raw environmental data were collected from the CHRONOS database, managed by Instituto de Fomento Pesquero, IFOP (Puerto Montt, Chile).

Samples were prepared according to the SMRTbell guide for sequencing on the PacBio Sequel II System. The genomic DNA isolated from 5 individuals collected from Puerto Montt was sequenced using SMRT sequencing according to the manufacturer’s protocols. SMRT sequencing yielded 882.1 Gb and 63 million long reads from 2 HiFi SMRT cells. The subreads N50 and average read lengths were 14,665 and 14,535 bp, respectively. The total HiFi reads yielded 3.7 million with an average quality of Q36 and Q35, respectively. Hi-C libraries were constructed from hemocyte cells using Phase Genomics’ Animal Hi-C kit and sequenced on Illumina’s Hiseq4000 (San Diego, CA, USA) platform to yield 253 million reads using the same DNA isolated for PacBio sequencing. Short-read sequencing libraries were prepared using an insert size of 150 bp obtained from 1 μg of genomic DNA after fragmentation, end-paired, and ligated to adaptors. The ligated fragments were fractionated on agarose gels and purified by PCR amplification to produce sequencing libraries. The method applied was like that previously published by Lieberman-Aiden et al. [44]. The PacBio and Hi-C Illumina DNA raw data were deposited in the NCBI Sequence Read Archive (SRA) repository, accession numbers SRR20593343 and SRR20966976, respectively.

Moreover, RNA libraries were constructed from hemocytes, digestive gland, gill, and mantle tissues for transcriptome sequencing to obtain whole-transcriptome profiling from the same mussels used for genome DNA sequencing. Additionally, twelve available Sequence Read Archive (SRA) transcriptomic data (GenBank accession number SRP261955), representing gills and mantle tissues collected from individuals of Cochamó and Yaldad mussel populations [37,38], were incorporated to analyze population-specific transcriptome profiles. Total RNA from three biological replicates (five total RNA extractions each) from each mussel population was extracted by the Trizol reagent method (Invitrogen, Waltham, MA, USA). The quality and integrity of extracted RNAs were measured in a Tape Station 2200 instrument (Agilent, Santa Clara, CA, USA), using the R6K Reagent Kit based on the manufacturer’s instructions. RNA samples with RNA integrity numbers (RIN) >9 were selected for the preparation of high-quality libraries using a TrueSeq Stranded mRNA LT Sample Prep Kit and sequenced in a HiSeq 4000 (Illumina, San Diego, CA, USA).

### 2.2. De Novo Genome Assembly and Hi-C Scaffolding of M. chilensis

Two HiFi single-molecule real-time cells in the PacBio Sequel platform yielded 53.8 Gb of high-quality DNA genome information. These long reads were assembled with the Hifiasm (v.0.19.3) package using default parameters [45]. For Hi-C scaffolding, reads were aligned to the primary draft assembly, following the manufacturer’s instructions [46]. Briefly, reads were aligned using BWA-MEM (v.0.7.17) [47] with the –5SP and –t 8 options specified and all other options default. The package SAMBLASTER (v.0.1.26) [48] was used to flag duplicates excluded from further analysis. Sequence alignments were filtered with SAMtools (v.1.17) [49,50] using the –F 2304 filtering flag to remove non-primary and secondary alignments. This step was conducted to remove alignment errors, low-quality alignments, and other alignment noise due to repetitiveness, heterozygosity, and other ambiguous assembled sequences. Finally, Phase Genomics’ Proximo Hi-C genome-scaffolding platform was used to create chromosome-scale scaffolds from the corrected assembly, according to Bickhart et al. [51].

### 2.3. Karyotype of M. chilensis

Metaphase plates of 24-h-post-fertilization larvae were used to obtain chromosomes from *M. chilensis*, according to Gallardo-Escárate et al. [52]. Briefly, antimitotic treatment with colchicine 0.05% solution was applied for 4 h. Then, the larvae were rinsed in clean seawater and immersed in a hypotonic solution (seawater:distilled water, 1:1) for 30 min. Finally, the larvae were fixed in modified Carnoy solution (methanol:acetic acid, 3:1). Chromosome spreads were obtained by dissociating larva tissue in acetic acid (50%), pipetting suspension drops onto slides preheated at 43 °C and air-dried according to Amar et al. [53]. A FISH experiment was performed to validate the physical localization of specific genes. Here, 28S rDNA was labeled following methods previously published [21]. Briefly, metaphase preparations were denatured at 69 °C for 2 min and hybridized overnight at 37 °C. Signal detection was performed using fluorescein avidin and biotinylated anti-avidin for the biotinylated probes, and mouse anti-digoxigenin, goat anti-mouse rhodamine, and rabbit anti-goat rhodamine for the digoxigenin-labeled probes. Fluorescent staining was carried out with 4,6-diamidino-2-phenylindole (DAPI) and mounted with Vectacshield antifading solution. Chromosome spreads were observed using an epifluorescent microscope Nikon Eclipse 80i (Minato-ku, Tokyo, Japan) equipped with a digital camera DS-5Mc.

### 2.4. Genome Annotation of M. chilensis

Our repeat annotation pipeline applied a combined homology alignment strategy, and de novo search to identify the whole-genome repeats. Tandem repeat was extracted using TRF (v.4.09.1) (http://tandem.bu.edu/trf/trf.html (accessed on 3 October 2021)) by ab initio prediction. The commonly used homolog prediction database Repbase (www.girinst.org/repbase (accessed on 3 October 2021)), employing RepeatMasker (v.4.1.5) (www.repeatmasker.org/ (accessed on 4 October 2021)) software and its in-house scripts (RepeatProteinMask) with default parameters was used to extract repeat regions. Ab initio prediction was used to build a de novo repetitive elements database by LTR_FINDER (v1.07) (https://github.com/xzhub/LTR_Finder (accessed on 4 October 2021)), RepeatScout (v.1.0.5) (www.repeatmasker.org/ (accessed on 4 October 2021)), and RepeatModeler (v.2.0.4) (www.repeatmasker.org/RepeatModeler.html (accessed on 3 October 2021)) with default parameters. Then, all repeat sequences with lengths >100 bp and gap ‘N’ less than 5% constituted the raw transposable element (TE) library. A custom library (a combination of Repbase and a custom de novo TE library processed by UCLUST (v.11) to yield a non-redundant library) was supplied to RepeatMasker for DNA-level repeat identification.

The structural annotation approach was applied to incorporate de novo, homolog prediction, and RNA-Seq-assisted predictions to annotate gene models. For gene prediction based on de novo, Augustus (v3.2.3), Geneid (v1.4), Genescan (v1.0), GlimmerHMM (v3.04), and SNAP (29 November 2013) were used in our automated gene prediction pipeline. Sequences of homologous proteins were downloaded from Ensembl/NCBI/others for homolog prediction. Protein sequences were aligned to the genome using TblastN (v2.2.26; E-value ≤ 1 × 10^−5^), and then the matching proteins were aligned to the homologous genome sequences for accurate spliced alignments with GeneWise (v2.4.1) software to predict the gene structure contained in each protein region. Finally, for RNA-seq data, transcriptome reads assemblies were generated with Trinity (v2.1.1) for the genome annotation. For the genome annotation optimization, the RNA-Seq reads from different tissues were aligned to genome fasta using Hisat (v2.0.4)/TopHat (v2.0.11) with default parameters to identify exons region and splice positions. The alignment results were inputted into Stringtie (v1.3.3)/Cufflinks (v2.2.1) with default parameters for genome-based transcript assembly. The non-redundant reference gene set was generated by merging genes predicted by three methods with EvidenceModeler (EVM v1.1.1) using PASA (Program to Assemble Spliced Alignment) terminal exon support and including masked transposable elements as input into gene prediction. Individual families of interest were selected for further manual curation.

Gene functions were assigned according to the best match by aligning the protein sequences to the Swiss-Prot database using Blastp (with a threshold of E-value ≤ 1 × 10^−5^). The motifs and domains were annotated using InterProScan70 (v5.31) by searching against publicly available databases, including ProDom, PRINTS, Pfam, SMRT, PANTHER, and PROSITE. Each gene’s gene ontology (GO) IDs were assigned according to the corresponding InterPro entry. We predicted the protein function by transferring annotations from the closest BLAST hit (E-value < 1 × 10^−5^) in the Swiss-Prot database and DIAMOND (v0.8.22)/BLAST hit (E-value < 1 × 10^−5^) in the NR database. We also mapped the gene set to a KEGG pathway and identified the best match for each gene.

Non-coding RNA annotations such as tRNAs were predicted using the program tRNAscan-SE (http://lowelab.ucsc.edu/tRNAscan-SE/ (accessed on 5 October 2021)). Since rRNAs are highly conserved, we chose relative species’ rRNA sequences as references and predicted rRNA sequences using Blast. Other ncRNAs, including miRNAs and snRNAs, were identified by searching against the Rfam database with default parameters using the infernal software (http://eddylab.org/infernal/ (accessed on 5 October 2021)). Additionally, lncRNAs were identified using the previously proposed pipelines [20,54,55].

### 2.5. Comparative Genomics between M. chilensis and M. coruscus

Syntenic relationships were explored among mussel species for which chromosome-level reference genomes are publicly available. Here, the analysis was performed between the two congeneric species *M. chilensis* (this study) and *M. coruscus* [28], where gene annotations were explored by MCScanX (v. 1.0) [56] implemented in the TBtools (v.1.115) package [57]. This approach detects groups of orthologous genes and compares their arrangement to identify colinear segments in the compared genomes. MCScanX was used to discover microsyntenic relationships, focusing on the local arrangement of genes near the syntenic blocks. The microsyntenic arrangement of genes identified by MCScanX was evaluated through GO analysis to identify the primary molecular function and biological processes enrichened for each genomic region where macromutations or chromosome rearrangements were detected.

Disseminated neoplasia is a disease horizontally transmitted by clonal cancer cells, which causes leukemia in mollusk bivalves [58,59]. The neoplastic cells gradually replace normal hemocytes leading to increased mortality, and the disease has been detected in 15 species of marine bivalve mollusks worldwide [60]. Notably, disseminated neoplasia has been observed among mussel species with varying epizootic prevalences. For instance, *M. trossulus* has shown high prevalences in some areas, whereas in *Mytilus edulis*, the prevalences are generally lower. Furthermore, *M. galloprovincialis* has been suggested as a species resistant to the disease in Spanish and Italian mussel populations [61]. This observation extends the relevance of exploring mussel species’ genetic features associated with disseminated neoplasia. Herein, the molecular characterization of Steamer-like elements in *M. chilensis* was conducted by cloning and the walking primer method according to Arriagada et al. [62]. The putative *M. chilensis* Steamer-like was scanned through twelve reference genomes assembled at chromosome level for Bivalvia: *Mytilus coruscus* (GCA_017311375.1), *Mytilus edulis* (GCA_019925275.1), *Dreissena polymorpha* (GCA_020536995.1), *Mercenaria mercenaria* (GCA_014805675.2), *Solen grandis* (GCA_021229015.1), *Ruditapes philippinarum* (GCA_009026015.1), *Pecten maximus* (GCA_902652985.1), *Pinctada imbricata* (GCA_002216045.1), *Crassostrea gigas* (GCA_902806645.1), *Crassostrea ariakensis* (GCA_020458035.1), and *Crassostrea virginica* (GCA_002022765.4). The putative long terminal repeat (LTR) sequences were identified using BLAST search, where open reading frames (ORFs) between flanking LTRs were detected. The identified Steamer-like elements were aligned using ClustalW and annotated based on a search of the NCBI Conserved Domain database. Amino acid sequences for the full-length Gag-Pol polyprotein region were aligned among the studied bivalve species. The Steamer element was reported for *Mya arenaria* (Accession AIE48224.1) and *M. chilensis* (this study). DNA sequence genealogy analysis was conducted to investigate horizontal transmission events among bivalve species. The maximum likelihood (ML) method was conducted on the SLEs loci localized in all the publicly available bivalve genomes assembled at the chromosome level.

### 2.6. Whole-Genome Transcript Expression Analysis in Two M. chilensis Populations

The transcriptomes of mussels collected from the Yaldad and Cochamó populations were analyzed using a hierarchical clustering approach to detect transcriptional similarities among tissues/populations. The differentially expressed transcripts compared to normalized expression values were visualized in a clustering heatmap and selected according to the identified cluster. For an optimal comparison of the results, k-means clustering was performed to identify candidate genes involved in specific gene expression patterns. The distance metric was calculated with the Manhattan method, where the mean expression level in 5-6 rounds of k-means clustering was subtracted.

Moreover, raw data from mussel tissues collected from the Yaldad and Cochamó populations were trimmed and mapped to the *M. chilensis* genome using CLC Genomics Workbench v22 software (Qiagen Bioinformatics, Aarhus, Denmark). Threshold values for transcripts were calculated from the coverage analysis using the Graph Threshold Areas tool in CLC Genomics Workbench v22 software. Here, an index denoted as chromosome genome expression (CGE) was applied to explore the whole-genome transcript expression profiling, according to Valenzuela et al. [63]. The CGE calculates the mean coverage of transcripts mapped into a specific chromosome region, comparing mussel populations and tissues. Specifically, the CGE index represents the percentage of the transcriptional variation between two or more RNA-seq data for the same locus. The transcript coverage values for each dataset were calculated using a threshold of 20,000 to 150,000 reads. A window size of 10 positions was set to calculate and identify chromosome regions differentially transcribed. This approach was used to visualize actively transcribed chromosome regions, identify differentially expressed genes, and observe tissue-specific patterns in the evaluated mussel populations. Finally, the threshold values for each dataset and the CGE index were visualized in Circos plots [64].

RNA-seq data analyses were carried out using the raw sequencing reads and mapped on the assembled genome by CLC Genomics Workbench v22 software (Qiagen Bioinformatics, Aarhus, Denmark) separately for each tissue/population. In parallel, de novo assembling was performed to evaluate PAVs and dispensable genes affecting the in-silico transcription analysis. The assembly was performed with overlap criteria of 70% and a similarity of 0.9 to exclude paralogous sequence variants. The settings were set as mismatch cost = 2, deletion cost = 3, insert cost = 3, minimum contig length = 200 base pairs, and trimming quality score = 0.05 using CLC Genomics Workbench v22. After assembly, the contigs generated for each data set were mapped on the genes annotated in the reference genome to evaluate genome coverage and detect PAV features. The analysis did not show bias putatively associated with PAVs between the analyzed mussel populations. Then, mRNA sequences annotated for the *M. chilensis* genome were used to evaluate the transcription level between mussel populations, where differential expression analysis was set with a minimum length fraction = 0.6 and a minimum similarity fraction (long reads) = 0.5. The obtained genes from each tissue/population were blasted to CGE regions to enrich the number of transcripts evaluated by RNA-Seq analysis. In addition, sequences were extracted near the threshold areas in a window of 10 kb for each transcriptome. The expression value was set as transcripts per million model (TPM). The distance metric was calculated with the Manhattan method, with the mean expression level in 5–6 rounds of k-means clustering subtracted. Finally, the Generalized Linear Model (GLM) available in the CLC software was used for statistical analyses and to compare gene expression levels regarding the log_2_ fold change (*p* = 0.005; FDR corrected).

Moreover, innate immunity in marine invertebrates may play an important role in speciation and environmental adaptation [65,66]. Herein, we investigate the immune-related genes associated with the Toll-like receptor (TLR) and apoptosis signaling pathways given that the functional annotation revealed that they were mainly enriched between the mussel populations analyzed. In addition, bioinformatic analyses were carried out using the CLC Genomics Workbench software to identify single nucleotide variants (SNV) from the transcriptomes sequenced for Yaldad and Cochamó. Candidate SNVs were called with the following settings: window length = 11, maximum gap and mismatch count = 2, minimum average quality of surrounding bases = 15, minimum quality of central base = 20, maximum coverage = 100, minimum coverage = 8, minimum variant frequency (%) = 35.0, and maximum expected variations (ploidy) = 2. Furthermore, the genotypes of DEGs were also identified for detecting putative genetic variations between mussel populations. Here, singleton, dispersed, tandem, proximal, and whole-genome duplication (WGD) gene events were evaluated using MCScanX. The amino acid changes and the zygosity proportions were also estimated in DEGs between the Yaldad and Cochamó populations.

### 2.7. GO Enrichment Analysis

Differentially expressed mRNA were annotated through BlastX analysis using a custom protein database constructed from GeneBank, KEGG, GO, and UniProtKB/Swiss-Prot. The cutoff E-value was set at 1 × 10^−10^. Transcripts were subjected to gene ontology (GO) analysis using the Blast2GO plugins included in the CLC Genomics Workbench v22 software (Qiagen Bioinformatics, Aarhus, Denmark). The results were plotted using the Profiler R package [67]. GO enrichment analysis was conducted to identify the most represented biological processes among protein-coding genes proximally located to the CGE regions. The enrichment of biological processes was identified using Fisher’s exact test tool of Blast2GO among the different tissues and mussel populations.

The Committee of Ethics, Bioethics, and Biosafety, University of Concepción, Chile, approved this project (CEB324-2022, September 2021).

## 3. Results and Discussion

### 3.1. Chromosome Genome Assembly of M. chilensis Using Proximity Ligation

With two HiFi single-molecule real-time cells in the PacBio Sequel platform, we generated 53.8 Gb of high-quality DNA genome information. This data comprised 63 million reads with a total length of 882 Gbp (Table 1). These long reads were assembled with the Hifiasm package using default parameters [45], yielding a primary assembly of 13,762 contigs equivalent to 2.19 Gb, with an N50 of 206 Mb. The genome size assembly made by Hifiasm was comparable with the previous genome size described for closely related species: 1.28 Gb for *M. galloprovincialis* [32], 1.57 Gb for *M. coruscus* [28], and 1.79 Gb for *Dreissena polymorpha* [27].

In vivo Hi-C is a technique that maps physical DNA–DNA proximity across the entire genome [68,69]. The method was introduced as a genome-wide version of its predecessor, 3C (chromosome conformation capture). It has been a powerful tool in chromosome-scale genome assembly of many animals in recent years [70,71]. In this study, Hi-C experiments and data analysis of hemocyte cells were used for the chromosome assembly of the blue mussel *M. chilensis*. Here, Phase Genomics (Seattle, WA, USA) prepared and sequenced two Hi-C libraries, resulting in ~20× coverage and ~253 million 150 bp paired-end reads (Table 1). The Hi-C analysis evidenced that 44.68% of high-quality reads showed intercontig signals or Cis-close position (<10 kbp on the same contig), and an additional 4.09% of sequence reads revealed a Cis-far conformation (>10 kbp on the same contig) (Table 2).

Hi-C reads were aligned using Bowtie version 1.3.1 [72] to order and orient the 13,762 contigs, and scaffolding was performed using Proximo (Phase Genomics, Seattle, WA, USA). We then applied Juicebox for visual inspection and manual correction [73]. We also manually removed 1894 scaffolds that were microbe-sized and disconnected from the rest of the assembly. Then, 11,868 contigs were used for the first chromosome-level high-quality *M. chilensis* assembly (Table 3).

The N50 and total genome length were calculated in 134 Mbp and 1938 Gbps, respectively. The *M. chilensis* genome provides a valuable genomic resource for research in mussel biology and for developing novel sustainable strategies in mussel aquaculture. The Hi-C data generated 14 chromosomes assembled with HiFi consensus long DNA reads (Figure 1B). The cytogenetic analysis performed for *M. chilensis* revealed a conservative karyotype for the *Mytilus* genus composed of 2n = 14 [21]. Physical localization of 28S-rRNA revealed two loci mapped in different submetacentric/subtelocentric chromosome pairs (Figure 1C), confirming the presence of major rDNA clusters subterminal to the long arms of two chromosome pairs reported in *M. edulis* and *M. galloprovincialis* [74]. Concerning genome assembly, the largest scaffold was assembled from 998 contigs with a total size of 173.3 Mb. Meanwhile, the smallest scaffold was 117.3 Mb, consisting of 744 contigs (Table 3). Notably, the number of contigs in the scaffolds was 11,868 (100% of all contigs in chromosome clusters, 86.24% of all contigs) and accounted for 1.93 Gbps of genome size (100% of all length in chromosome clusters, 88.43% of all sequence length). The completeness of genome assembly was assessed by the single-copy ortholog set (BUSCO, V5.3.2) [75]. The results showed the following BUSCO scores: (i) Eukaryota Odb10; C:94.1% (S:72.9%, D:21.2%), F:3.1%, M:2.8%, and n:255. (ii) Metazoa Odb10; C:95.1% (S:75.5%, D:19.6%), F:2.5%, M:2.4%, and n:954. (iii) Mollusca Odb10, C:85% (S:70.1%, D:14.9%), F:3.6%, M:11.4%, and n:5295.

### 3.2. Genome Annotation of M. chilensis

**Figure 2:**
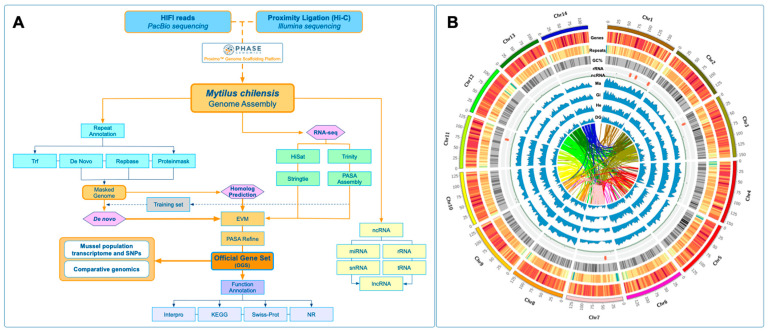
The native blue mussel *Mytilus chilensis* genome. (**A**) Workflow of de novo whole-genome sequencing project and annotation for *M. chilensis*. The rectangles indicate the steps of the primary data processing, and the arrows indicate output or input data. Pink diamonds indicate the combined strategy based on homolog prediction, de novo, and RNA-seq-assisted prediction. (**B**) The Circos plot shows the genomic features for the 14 pseudo-chromosomes. From the outer to the inner circle: gene density, repeat density, GC content, rRNA localization, and ncRNAs. The transcriptome expression for mantle (Ma), gills (Gi), hemocytes (He), and digestive gland (DG) are shown as light blue profiles. Chromosome syntenies are represented in different colors according to each ideogram. The chromosome size is shown in the Mb scale.

The genome assembly was annotated using de novo and protein- and transcript-guided methods (Figure 2A). The first step of the annotation process was to identify the DNA repeats through the *M. chilensis* genome. Repetitive elements and non-coding genes in the blue mussel genome were annotated by homologous comparison and ab initio prediction. RepeatMasker [76] was used for homologous comparison by searching against the Repbase database [77] and RepeatModeler [78]. According to these analyses, about 1.1 Gbps of repeat sequences were annotated, which accounted for 56.73% of the whole genome. Herein, DNA transposons, LINE, and LTR transposable elements were identified (Table 4). Useful genome information for population genetic studies is the identification of simple sequence repeats (SSRs) or microsatellites. The mining of SSRs revealed that the *M. chilensis* genome has 548,360 SSR sequences, where 9% and 6% of the SSR loci were annotated for each mussel chromosome (Figure S1). The most frequent SSR motif was the tetranucleotide, followed by the dinucleotides, accounting for 206,103 and 197,700 repeats, respectively. The entire SSR sequences accounted for 0.35% of the whole genome. The development of SSR markers offers a shortcut to assessing genetic diversity, which can potentially be applied in food authentication and genetic traceability for mussel species [79,80,81].

### 3.3. Protein-Coding Gene Prediction and Functional Annotation in the M. chilensis Genome

For the identification of protein-coding genes, de novo, homolog prediction, and RNA-seq evidence were used as the training set (Figure 2A). For homologous predictions, the protein sequences from *Crassostrea gigas*, *Mytilus galloprovincialis*, *M. coruscus*, and *Dreissena polymorpha* genomes were extracted using the respectively published references and aligned against the blue mussel genome using TBLASTN (E-value < 1 × 10^−5^) (Table 5). The gene sequence structure of each candidate gene and previously mentioned tools were used to predict protein-coding genes. Finally, a non-redundant reference gene set was generated using the EvidenceModeler (v.2.0) (EVM) and PASA2 tools (v.2.5.2) (Figure 2A).

Taken together, 34,530 protein-coding genes were identified with a 6531 bp average transcript length, 1377 bp average CDS length, 4.92 average of exons per gene, and 1377 and 1316 average length of exons and introns, respectively (Table 6). Additionally, 516 tRNAs were predicted using tRNAscan-SE, and 143 rRNA genes were annotated using RNAmmer. For non-coding RNAs with putative regulatory roles, 1365 miRNAs and 43,011 long non-coding RNAs were identified and annotated within the *M. chilensis* genome (Table 7). For functional annotation, the predicted proteins within the blue mussel genome were searched by homology against seven databases: Swiss-Prot, Nr, Nt, KEGG, eggnog, GO, and Pfam (Figure 2A). Overall, 70.45%, 73.01%, 8.98%, 64.94%, 80.57%, 33.61%, and 96.33% of genes matched entries in these databases, respectively. A total of 34,530 genes (100%) were successfully annotated by gene function and conserved protein motifs (Table 8). The genomic features annotated for the native blue mussel *M. chilensis* were displayed using a Circos plot [64]. Herein, this graphical representation shows the primary genomic features for the 14 chromosomes. Specifically, gene density, repeat density, GC content, rRNA localization, and ncRNAs were plotted. The transcriptome expression profiles for the mantle, gills, hemocytes, and digestive gland tissues were also displayed in connection with the syntenic blocks (Figure 2B).

### 3.4. Comparative Genomics

Smooth-shelled blue mussels of the genus *Mytilus* represent a model group because of their cosmopolitan distribution, socioecological importance, and intriguing evolutionary history. This taxon provides new insights into the process of speciation and how hybridization and introgression can be one of the biggest threats to global mussel biodiversity [82]. A survey of single nucleotide polymorphisms (SNPs) on southern hemisphere blue mussels has provided a new layer for understanding their biology, taxonomy, and phylogeography [83,84]. However, SNP markers cannot be applied as a single tool to evidence chromosome rearrangement events during the *Mytilus* evolution. Here, whole-genome sequencing in smooth-shelled blue mussels and relative bivalve species is a priority for global mussel aquaculture, biosecurity, and conservation.

**Figure 3:**
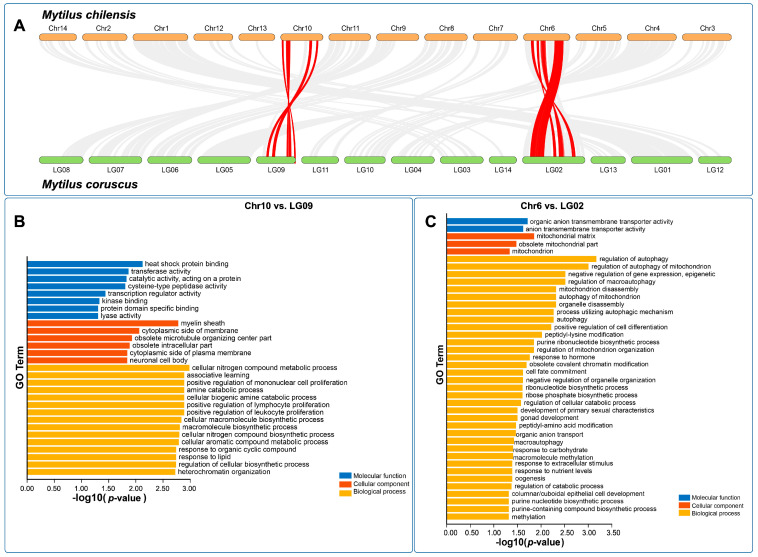
(**A**) Whole-genome macrosyntenic relationships between *M. chilensis* and *M. coruscus.* Orthologous relationships among mussel chromosomes are highlighted in grey, and the orthologous relationships between Chr6 and LG02, and Chr10 and LG09 are highlighted in red. (**B**,**C**) Gene ontology term annotated for the syntenic chromosome regions identified by MCScanX for Chr6 vs. LG02, and Chr10 vs. LG09. The *x*-axis indicates the negative log_10_ (*p*-value).

With the aim of exploring genomic rearrangements in *Mytilus*, the reported reference genomes for *M. coruscus* and *M. chilensis* were analyzed. Of the 34,530 predicted genes from the *M. chilensis* genome, 18,758 (54.32%) were found in syntenic collinear blocks after being compared with the *M. coruscus* genome (Figure 3A). These syntenic blocks consisted of 671 alignments with a minimum of 5 genes per block. The number of alignments per chromosome ranged from 27 on chromosome 13 to 69 on chromosome 3. Chromosomes with higher genes in collinear blocks were chromosomes 1, 4, and 6, with 1227, 1091, and 1088 genes, respectively. Blocks with less than five genes or E-value < 1 × 10^−5^ were discarded from this analysis. Most collinear blocks were located at the same pair of chromosomes between the two genomes. For example, *M. chilensis* Chr1 had only syntenic blocks with LG01 from *M. coruscus* in the same order. However, chromosomes 6 and 10 from *M. chilensis* had collinearity with chromosomes LG09 and LG02 in *M. coruscus* but were orientated as two inversed blocks per pair of chromosomes (red lines in Figure 3A and Figure S2). The genes in these alignments from inversed blocks were extracted, blasted, and gene ontology terms were identified. Enrichment analyses from GO terms were obtained from Chr10, and LG09 inversed blocks and Chr6 and LG02 pair of chromosomes (Figure 3B,C). Most molecular function-enriched GO terms in the Chr10 and LG09 pair were associated with heat shock protein (HSP) binding. By contrast, in the Chr6 and LG02 pair, most of the enriched GO terms were associated with the mitochondria and biological processes related to autophagy or regulation of gene expression by epigenetic changes. Notably, chromosome rearrangements have been associated with adaptative genetic traits in marine organisms [85], where specific architectural proteins such as HSPs may have distinct roles in establishing 3D genome organization [86].

### 3.5. Comparative Analysis of Steamer-like Elements in Bivalvia

**Figure 4:**
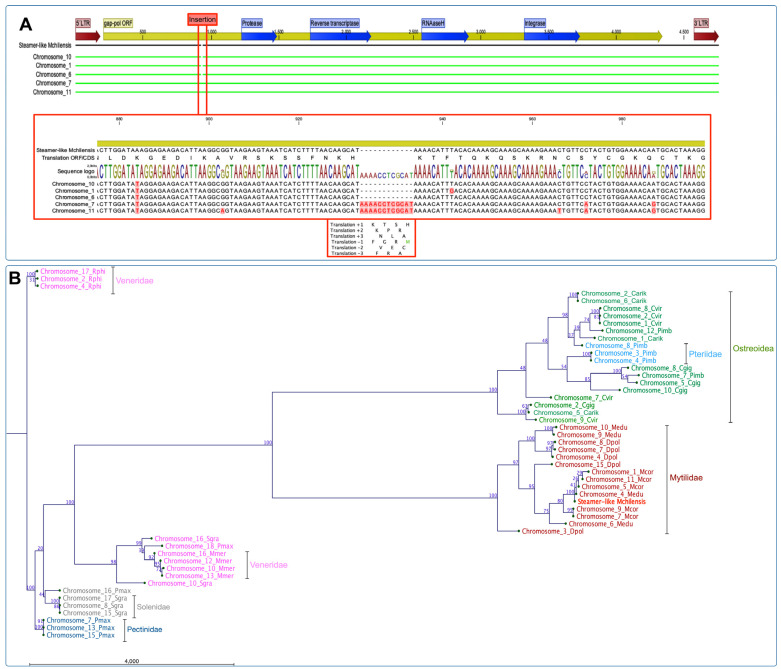

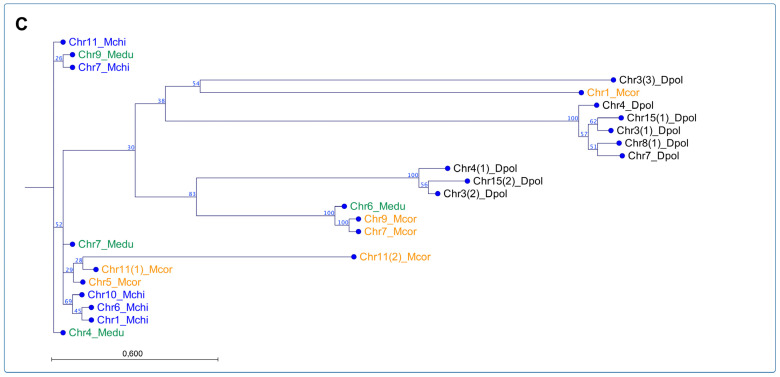
Molecular characterization of Steamer-like elements (SLE) in the *M. chilensis* genome and phylogenetic analysis using publicly available reference genomes assembled at chromosome level for bivalve species. (**A**) Alignment of five SLE copies localized in chromosomes 1, 6, 7, 10, and 11. All the SLEs are flanked by two LTRs (5′ and 3′) containing the Gag-Pol ORFs and the domains annotated for protease, reverse transcriptase, RNAaseH, and integrase. An insertion composed of 12 nucleotides (933^934 position) was found in chromosomes 7 and 11. The detailed alignment and the translation for the nucleotides inserted are highlighted in the red box. (**B**) Maximum likelihood (ML) phylogenetic tree of nucleotide sequences from SLEs found in eleven reference genomes for Bivalvia. Colored chromosomes and numbers indicate the SLE genome localization and the bivalve species, respectively. The species analyzed were: Veneridae (pink) *Ruditapes philippinarum* (Rphi) and *Mercenaria mercenaria* (Mmer); Solenidae (grey) *Solen grandis* (Sgra); Pectinidae (blue) *Pecten maximus* (Pmax); Ostreidae (green) *Crassostrea gigas* (Cgig), *C. virginica* (Cvir), and *C. ariakensis* (Caria)*;* Pteriidae (light blue) *Pinctada imbricata* (Pimb); and Mytilidae (red) *Mytilus coruscus* (Mcor), *Mytilus edulis* (Medu), and *Dreissena polymorpha* (Dpol). (**C**) ML analysis of SLEs identified in *M. edulis* (orange), *M. coruscus* (orange), *D. polymorpha* (black), and *M. chilensis* (blue) chromosomes.

To explore the gene expansion of retrotransposon elements among representative species from Bivalvia, we primarily characterized the Steamer-like elements (SLEs) in *M. chilensis* using the approach described by Arriagada et al. [62]. The analysis evidenced that the genome of *M. chilensis* contains five copies of SLEs distributed in chromosomes 1, 6, 7, 10, and 11. The alignment showed that all SLE copies are flanked by two LTRs (5′ and 3′) containing the Gag-Pol ORFs and the domains annotated to protease, reverse transcriptase, RNAaseH, and integrase. Notably, an insertion composed of 12 nucleotides at position 933^934 was exclusively found in chromosomes 7 and 11. The translation for the inserted nucleotides suggests four amino acids, K, T, S, and H, in a positive orientation. However, the translation evaluated in the reading frame (−1) evidenced a methionine localized before the RNAaseH coding gene (Figure 4A).

Furthermore, the phylogenetic analysis using publicly available reference genomes assembled at chromosome level for eleven bivalve species using maximum likelihood (ML) revealed a six-chromosome cluster composed of bivalves belonging to the families Veneridae, Solenidae, Pectinidae, Ostreidae, Pteriidae, and Mytilidae (Figure 4B). The phylogenetic-reconstruction-rooted SLEs were found in three chromosomes (2, 4, and 17) from *R. philippinarum.* The other Veneridae member, *M. mercenaria* showed a cluster of four chromosomes (10, 12, 13, and 16) and related to two chromosomes of *S. grandis* (10 and 16). This last species formed a unique cluster composed of three chromosomes (8, 15, and 17), similar to *P. maximus*, with three chromosomes. Concerning the mussel and oyster genomes assembled at the chromosome level, the phylogenic analysis revealed two main clusters composed of species belonging to Ostreida and Mytilidae, where the first taxon was comprised of the Ostreidae and Pteriidae families. Herein, one cluster was rooted with three SLE sequences from *C. virginica*, *C. gigas*, and *C. ariakensis* located on chromosomes 9, 2, and 5, respectively. The second major cluster was composed of SLEs annotated in chromosomes from Ostreidae and Pteriidae, where *C. virginica* chromosomes were closely related to *P. imbricata*. The third cluster was observed containing three SLE sequences from *C. virginica* and *C. ariakensis*: chromosomes 1, 2, 8, and 1, 2, and 6, respectively (Figure 4B). The analysis of the Mytilidae family revealed two primary clusters comprising SLEs located in chromosomes from *M. edulis* and *D. polymorpha*, and *M. coruscus*, *M. chilensis*, respectively (Figure 4B). This last cluster grouped five chromosomes from *M. coruscus* (Chr. 1, 4, 5, 7, and 11), and two from *M. edulis* (Chr. 4 and 6). The Steamer-like sequence characterized for *M. chilensis* was also observed in this cluster. Finally, a detailed analysis of the three mussel species reported with genome assemblies at the chromosome level was conducted (Figure 4C). Notably, a rooted cluster comprising chromosomes 7, 11, 9 for *M. couscous*, and 4 and 6 for *M. edulis* were closely related. Herein, two primary clusters of SLEs located in chromosomes from *M. chilensis*, *M. edulis*, and *M. coruscus* were observed. The analysis suggested that the SLEs identified on *M. chilensis* chromosomes are closely related to the SLEs annotated on chromosomes 9 and 4 in *M. edulis*; meanwhile, the SLEs located in chromosomes 1, 5, and 11 in *M. coruscus* were also identified in the same chromosome cluster. The second main cluster observed comprised exclusively SLEs annotated in *D. polymorpha* chromosomes, except the SLE copies identified in chromosomes 9 and 10 of *M. edulis*. Interestingly, the SLEs annotated in chromosome 9 from *M. edulis* are shared among the three primary clusters analyzed, suggesting putative translocation gene events in Mytilidae.

Overall, the phylogenic relationships of SLEs revealed that the reported bivalve genomes comprise between 3 and 6 loci. A lower number of SLEs was found in Solenidae, Pectinidae, and Veneridae, followed by Mytilidae. A higher number of SLE loci was observed in genomes belonging to the Ostreida order. As far as we know, the evolution of the bivalve chromosomes has mainly been studied using cytogenetic techniques combining molecular probes on candidate genes to detect genome rearrangements that drive the speciation process [87,88,89]. However, the availability of reference genomes assembled at the chromosome level opens new perspectives for exploring molecular evolution in several taxonomic orders through gene collinearity analysis. The study by Yang [28] highlighted putative chromosome rearrangements among the king scallop *Pecten maximus*, the blood clam *Scapharca broughtonii*, the hard-shelled mussel *Mytilus coruscus*, the pearl oyster *Pinctada martensii*, and the Pacific oyster *Crassostrea gigas* genomes. Notably, the chromosome synteny illustrated that large-scale rearrangements are common events between the scallop and oyster but scarce between the scallop and mussel genomes. The reported evidence suggested that almost all the chromosome rearrangements between the mussel and oyster genomes are different, implicating independent chromosome fusion events. The SLE loci identified in all the genomes analyzed in the current study suggest that SLEs are relatively conserved in chromosome position for some taxa. For instance, the SLE loci in Veneridae, Pectinidae, and Solenidae appear to be associated with chromosomes 10, 13, 12, and 16. This sharing characteristic can reflect common genetic events during the evolution of these taxonomical groups. Similarly, the Ostreidae and Mytilidae families share SLE loci annotated to chromosomes 1, 2, 8, and 10. The detailed analysis of SLEs in Mytilidae shows that the transposon identified in *M. chilensis* was shared between *M. edulis* and *M. coruscus*, where SLEs in *D. polymorpha* appear to be more phylogenetically distant than *Mytilus* species. Interestingly, the mutation identified on the SLEs localized in the *M. chilensis* genome (insertion of twelve nucleotides), specifically on chromosomes 7 and 11, was shared with the SLE annotated on chromosome 9 in *M. edulis*. This cumulative evidence reveals diverse chromosome rearrangements, reflecting a complex evolutionary history of bivalve chromosomes.

### 3.6. The Marine Environment of M. chilensis Populations

**Figure 5:**
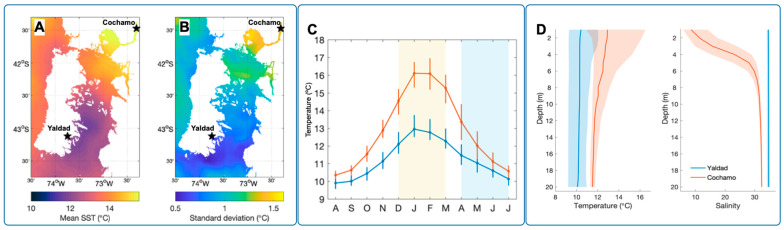
Temporal and spatial variability around Chiloé island, and at the Yaldad and Cochamó sites, over the past two decades. (**A**,**B**) Maps correspond to mean SST computed for December 2017–March 2018 and the corresponding standard deviations. Stars indicate the location of sampling sites. (**C**) Monthly medians computed from data in (**A**), showing the first and third quartiles as error bars; note that the sequence of months shown on the *x*-axis begins in August and ends in July. Shaded areas indicate summer (yellow) and fall–winter (blue) periods. (**D**) In situ measurements for temperature (°C) and salinity (PSU) from 0 to 20 m of depth. The blue and red lines for (**C**,**D**) represent the data collected from Yaldad and Cochamó, respectively.

Temporal and spatial variability of sea surface temperature (SST) around Chiloé island and at Yaldad and Cochamó were analyzed over the past two decades. The oceanographic variability for the location studied was analyzed from remote sensing data and in situ measurements (Figure 5A–D). Here, the daily time series of SST extracted from satellite-derived data for both sites evidenced high surface temperature variability between Yaldad and Cochamó, where this last location was constantly higher throughout the year (Figure S3). Notably, the monthly medians computed from the SST time series showed that the main differences were observed during the austral summer from December to March. During the winter, the oceanographic variability was less pronounced, showing temperatures between 13 °C and 10 °C from April to July (Figure 5C).

Furthermore, in situ data were collected from June 2017 to May 2018, exhibiting significant differences in both locations for temperature and salinity between 0 and 20 m of depth. Interestingly, the oceanographic survey revealed a pronounced vertical stratification with higher temperatures and lower salinity in Cochamó compared with Yaldad (Figure 5D). These observations support the idea that two oceanographically different zones exist in the inner sea of Chiloé Island. In this northern area, mussels from Cochamó and Yaldad were sampled for the current study. Taken together, we can hypothesize that the mussels inhabiting Cochamó are significantly more exposed to environmental stress than the Yaldad mussel population. To date, there are few studies exploring how population genetic variation is related to, or caused by, the marine environmental variation in mussel populations. Notably, a study conducted by Wenne et al. [90] examined the genetic differentiation of native populations of *M. galloprovincialis* throughout its entire geographic range in the Mediterranean Sea, the Black Sea, and the Sea of Azov using 53 SNP loci. The results indicated that 7 of the 13 environmental variables explained significant variation in population-specific SNP locus allele frequencies. These seven variables explained 75% of the variation in the SNP dataset, suggesting that a complex mix of environmental variables contributes to the genetic variation in *M. galloprovincialis* populations in the Mediterranean Sea.

### 3.7. Whole-Genome Transcript Expression Analysis in Two M. chilensis Populations

**Figure 6:**
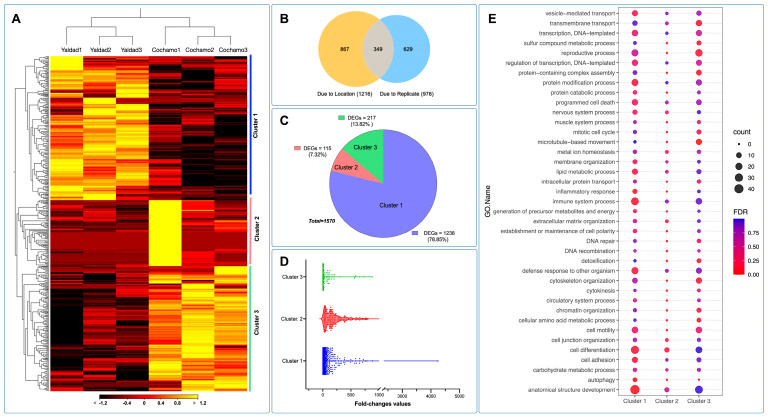
Population-specific transcriptome analysis in the blue mussel *M. chilensis*. (**A**) Transcriptome patterns of coding genes analyzed in gills from Yaldad and Cochamó populations. Three replicates were evaluated from each experimental group. The heatmap was based on transcripts per million (TPM) calculation and hierarchical clustering on Manhattan distances with average linkage (fold change ≥ |4|; FDR = 0.05). Yellow colors mean upregulated coding genes and black represents downregulated genes. (**B**) Venn diagram showing shared and unique genes expressed among the locations and replicates. (**C**) Pie chart showing the number of differentially expressed genes (DEGs) annotated for expression clusters 1, 2, and 3 between the Yaldad and Cochamó populations. (**D**) Fold-change values observed for DEGs identified in each evaluated cluster. (**E**) GO enrichment of cluster-specific genes (*p*-value < 10–16; |fold-change| > 4) annotated for key biological processes differentially expressed. The *y*-axis indicates the GO term, and the *x*-axis indicates the clusters identified in (**A**). The color bar indicates the enrichment analysis estimated by FDR (false discovery rate) < 0.05 for the DEGs (fold change > 4, *p* < 0.01). The bubble size indicates the number of genes associated with the given GO term.

The transcriptome profiling among mussels collected during the austral summer in 2019 from Yaldad and Cochamó evidenced three primary transcriptional clusters. Herein, gene cluster 1 was highly expressed in the gills of mussels exposed to the Yaldad marine conditions; meanwhile, gene clusters 2 and 3 were highly expressed in individuals collected in Cochamó or mussels exposed to estuarine conditions (Figure 6A). Notably, the RNA-seq from individuals collected as Cochamo1 (replicate) showed a highly expressed gene cluster, indicating a wide transcriptome variation among mussels from this population. The RNA-seq analysis was performed with the mRNA sequences annotated on the *M. chilensis* genome. Herein, it is essential to note that in mussel species, specifically in *M. galloprovincialis*, the phenomenon of presence–absence variation (PAV) has been described. This fact means that PAVs can bias the analyses of transcriptome profiles in the studied mussel populations. We previously conducted a de novo assembling for the RNA-data sets sequenced from Yaldad and Cochamó populations. The results showed that the number of genes with expression values >1 (total gene reads) did not show statistical differences between both mussel populations. For instance, Yaldad and Cochamó mussels showed 25,086 ± 215 and 25,344 ± 212 (three replicates per population), respectively, of expressed genes in gill tissue (*p*-value = 0.98). Collectively, between 72.6% and 73.3% of the annotated genes in the *M. chilensis* genome were transcriptionally active in gills independently of the population analyzed.

The evaluation of differentially expressed genes (DEGs) showed that the main factor of differences in the number of DEGs was the population rather than the replicates assessed (Figure 6B). The proportion of DEGs evaluated among the gene clusters revealed that cluster 1, highly expressed in Yaldad, accounted the 78.85% of the total DEGs analyzed. Clusters 2 and 3 are primarily characterized by high transcription values in the Cochamó population, evidenced by 7.32% and 13.82% of DEGs, respectively. The total number of DEGs analyzed was 1570 (Figure 6C). Notably, the fold-change values estimated among the replicates and populations revealed high values in gene transcriptional cluster 1, compared with clusters 2 and 3 where the fold-change values were significantly lower (Figure 6D). The functional analysis showed that cluster 1 was enriched by GO terms related to protein modification processes, programmed cell death, immune system processes, defense response, cell differentiation, and anatomical structure development (Figure 6E). Clusters 2 and 3 were less enriched, revealing significant GO terms for transmembrane transport, reproductive processes, protein-containing complex assembly, microtube-based movement, cytoskeleton organization, chromatin organization, and metabolic process (Figure 6E).

**Figure 7:**
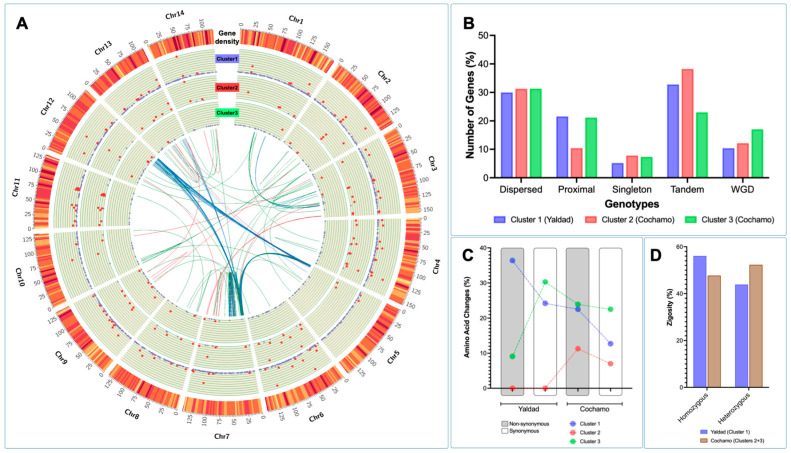
Genetic polymorphisms annotated in differentially expressed genes (DEGs) analyzed between the Yaldad and Cochamó mussel populations. The evaluation of DEGs was performed by cluster transcriptome analysis. (**A**) Circos plot showing DEGs identified in the three analyzed clusters. From outer to the inner circle: gene density, DEG cluster 1, DEG cluster 2, DEG cluster 3, and syntenic relationships between DEGs (each color line represents the cluster analyzed). Red dots represent DEGs with fold-change values > |100|, and purple dots represent fold-change values < |10|. (**B**) Genotypes of DEGs identified in *M. chilensis* populations according to the transcription cluster analysis. Singleton means that the gene is single-copy, which should not be the type of members of gene families. Dispersed means that the gene might arise from transposition. Tandem means that genes were repeatedly duplicated. Proximal means that the gene might arise from small-scale transposition or from tandem duplication and insertion of some other genes. Whole-genome duplication (WGD) means the gene might arise from a chromosome duplication region. The analysis was carried out using *MCScanX*. (**C**) Amino acid change proportions (%) between the Yaldad and Cochamó populations. The non-synonymous and synonymous were annotated for the DEGs selected for each mussel population according to the cluster analysis. (**D**) Zygosity proportion (%) estimated for each mussel population. Cluster 1 (Yaldad) is represented by blue bars, and clusters 2 and 3 (Cochamó) are displayed in brown bars.

The cluster gene expression analysis was used to identify genetic polymorphisms annotated in differentially expressed genes (DEGs) between the Yaldad and Cochamó mussel populations. The DEGs were evaluated by cluster transcriptome analysis displayed using a Circos plot to visualize specific loci where DEGs were highly transcribed. The fold-change values calculated showed high transcription levels in clusters 1 and 2 through all chromosomes scanned (see red dots in Figure 7A). Congruently with the previous RNA-seq results in this study, the highest fold-change values were observed in DEGs annotated in cluster 1 (Yaldad population). By contrast, cluster 3 showed a small number of DEGs with high fold-change values. Notably, the physical mapping of DEGs on chromosomes evidenced specific transcriptome patterns, revealing genes differentially expressed through the mussel genome exposed to the marine environment. The synteny analysis for DEGs showed a marked pattern among chromosomes 5, 7, and 12 for cluster 1; meanwhile, the synteny observed for the DEGs annotated in clusters 2 and 3 revealed a wide distribution along the *M. chilensis* genome (Figure 7A). Interestingly, the analysis carried out to detect macro-genome mutation in gene families between the Yaldad and Cochamó populations evidenced a similar number of dispersed genes, suggesting that those might arise from transposition. Tandem or repeatedly duplicated genes were observed with a low proportion in cluster 3 (Cochamó); meanwhile, the proximal genes showed a similar proportion to cluster 1 (Yaldad). These results might suggest small-scale transposition or duplication/insertion events. An interesting finding was observed for whole-genome duplication (WGD). The primary proportion was evidenced in cluster 3 (Cochamó), compared with clusters 1 and 2 from the Yaldad population (Figure 7B). Furthermore, the bioinformatic analysis conducted for detecting amino acid changes (AAC) in DEGs showed that 38% of non-synonymous AAC were identified in mussels collected from Yaldad. By contrast, the main proportion of synonymous AAC was detected in mussels exposed to Cochamó’s estuarine conditions (Figure 7C). Notably, the analysis performed for DEGs annotated in cluster 2 did not show non-synonymous and synonymous AAC in mussels collected from Yaldad. Finally, evaluating the zygosity proportion estimated for each mussel population evidenced an inverse pattern between both populations. The Yaldad cluster was higher in the homozygous proportion than Cochamó, where heterozygous AAC were detected in a higher proportion (Figure 7D).

**Figure 8:**
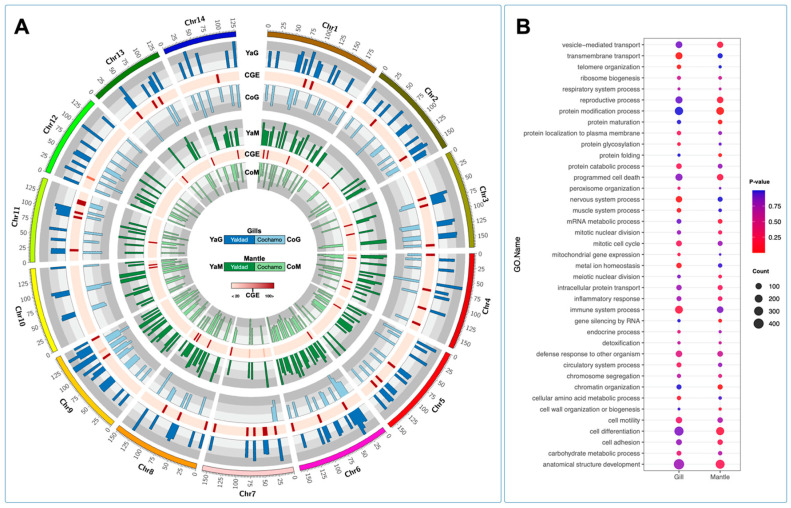
Chromosome–genome transcription in *M. chilensis* tissues collected from the Yaldad and Cochamó populations. (**A**) Circos plots showing the threshold values for transcriptional differences between locations (Yaldad/Cochamó) and tissues (gills/mantle). The differences were estimated through the CGE index. Heatmap in red shows the variation in gene expression from high to low differences. (**B**) GO enrichment of tissue-specific genes (*p*-value < 10–16; |fold-change| > 5) annotated for key biological processes differentially expressed. The *y*-axis indicates the GO term, and the *x*-axis indicates the tissues analyzed. The color bar indicates the enriched factor. The bubble size indicates the number of GO terms.

To explore the transcriptome signatures between the Yaldad and Cochamó mussel populations, we applied the genome chromosome expression (CGE) approach to test differences among tissues and individuals through the *M. chilensis* genome. The CGE analysis revealed high differences among chromosome regions, where the gill tissue was more modulated than the mantle tissue (Figure 8A). Interestingly, there are some levels of congruence among the CGE annotated for both mussel populations. We conducted a gene ontology enrichment analysis using this finding from genes identified by CGE analysis. The results evidenced that gill transcriptomes displayed functional processes associated with transmembrane transport, protein catabolic, nervous system, and metal homeostasis. Notably, immune system process GO terms were highly enriched in gills. Moreover, the chromosome region differentially expressed in mantle tissue revealed that the reproductive process, protein modification process, cell differentiation, anatomical structure development, and gene silencing by RNA were mainly annotated (Figure 8B). Taken together, the results reported in this study are highly congruent with the previous study conducted by Yévenes et al. [38], through the transcriptome responses of *M. chilensis* collected in ecologically different farm-impacted seedbeds.

**Figure 9:**
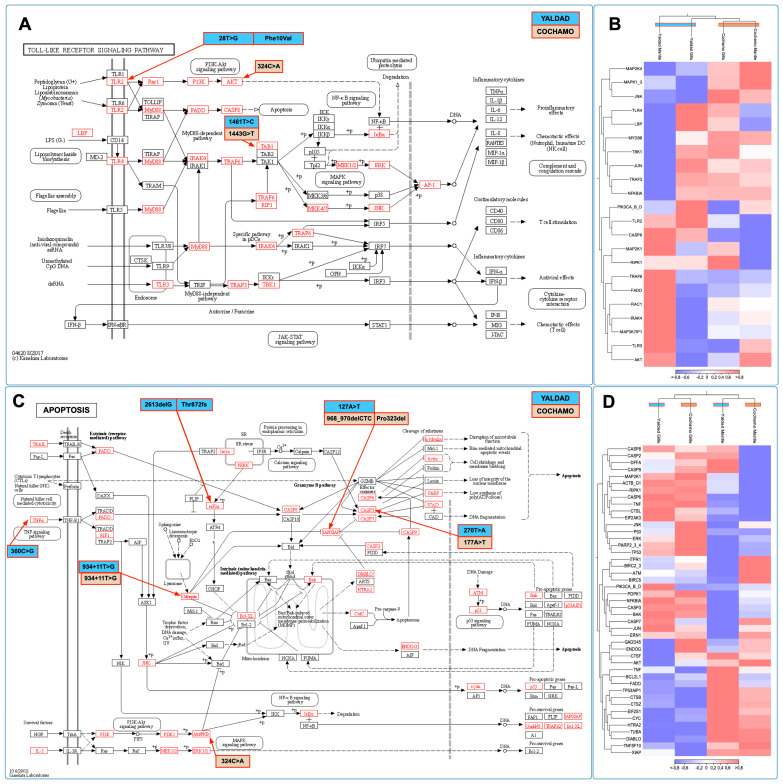
Transcriptome response of the immune system by KEGG pathway analysis and single mutation variant detection between *M. chilensis* populations. (**A**) Toll-like receptor (TLR) and (**C**) apoptosis signaling pathway comparisons between mussels from Yaldad and Cochamó. Single nucleotide polymorphisms and amino acid changes are shown in blue and brown boxes according to the mussel population. Identified gene families on the KEGG pathways are marked with red in the *M. chilensis* genome. (**B**,**D**) Transcriptome patterns of coding genes analyzed in gills and mantle tissue from the Yaldad and Cochamó populations for TLR and apoptosis-related genes. Three replicates were evaluated from each experimental group. The heatmap was based on transcripts per million (TPM) calculation and hierarchical clustering on Manhattan distances with average linkage. Red colors mean upregulated coding genes and blue colors represent downregulated genes.

The cumulative findings of this study suggest that the immune system was primarily modulated between mussels exposed to the Yaldad and Cochamó environmental conditions. With the aim of exploring the transcription profiling of immune-related genes, we selected two KEGG pathways annotated in the *M. chilensis* genome (Figure 9). Herein, Toll-like receptor signaling pathway and apoptosis were analyzed in terms of transcription activity and single nucleotide variation (SNV) between mussel populations. Notably, a non-synonymous SNV was detected on the *TLR2* gene (28T>G) in individuals collected from the Yaldad population. The translation evidenced an amino acid change from phenylalanine to valine at position 10 in the ORF (Phe10Val) (Figure 9A). The analysis also evidenced SNV on genes such as *AKT* and *TAB1*, where no amino acid changes were detected. The transcriptome profiling for the TLR pathway evidenced a high modulation of genes such as *TLR3*, *AKT*, *TRAFF6*, *FADD*, *IRAK4*, and *RAC1* in mussels collected from Yaldad. Interestingly, mitogen-activated protein kinases (*MAP2K* and *MAPK1*) and c-Jun N-terminal kinase (*JNK*) were differentially expressed, suggesting putative roles related to stress signaling pathways (Figure 9B). Furthermore, the apoptosis pathway revealed two SNV localized in eukaryotic initiation factor 2 α (*EIF2α*) and inhibitor of apoptosis (*IAP*) in mussels sampled from the Yaldad and Cochamó populations, respectively (Figure 9C). The 2613delG in the *EIF2α* gene produces a frameshift at the Thr872; meanwhile, the 968_970delCTC localized in the IAP gene produces a deletion of proline at position 323. The transcriptome profiling of apoptosis-related genes showed a conspicuous differentiation between gills and mantle tissue, where three primary gene expression clusters were identified (Figure 9D).

Notably, genes such as *P53*, *ERK*, *TP53*, *PARP2*, and *JNK* were highly expressed in gill tissue. The gene expression analysis in mantle tissue evidenced high transcriptional activity in genes related to the intrinsic (mitochondria-mediated) pathway, such as the B-cell lymphoma (*BCL*) gene and the second mitochondria-derived activator of caspase (*DIABLO*) gene. Concerning the mitogenome of *M. chilensis*, it was previously reported by Gaitán-Espitia et al. [34] and Śmietanka and Burzyński [91], evidencing a genome size of 16,748bp and structurally identical to the northern hemisphere *M. edulis* and *M. galloprovincialis* mitogenomes. Furthermore, the putative adaptive contribution of the mitochondrial genes was recently reported by Yevenes et al. [37]. The RNA-Seq analysis detected differences in the number of upregulated mitogens between individuals from Cochamó and Yaldad, some being tissue-specific (*ND4L* and *COX2*). Several monomorphic location-specific mitochondrial genetic variants were detected in samples from Cochamó and Yaldad, representing standing genetic variability to optimize mitochondrial functioning under local habitats. Overall, these mitochondrial transcriptomic differences reflect the impact of environmental conditions on the mitochondrial genome functioning and offer new markers to assess the effects of habitat translocations on mussel fitness, a routine industry practice. Likewise, these mitochondrial markers should help monitor and maintain population differences in this keystone and heavily exploited native species.

Further functional studies will be conducted to validate the association between single nucleotide polymorphisms and the fitness traits observed or how the translocation process associated with aquaculture activity can evolve with the loss of locally adapted alleles. Interestingly, a recent transplant experiment reported by Jahnsen-Guzmán et al. [92] demonstrated that *M. chilensis* individuals are adapted to the subtidal environment (4 m depth), as they exhibit significantly higher fitness (growth and calcification rates) than those transferred to the intertidal environment (1 m depth), which showed increased metabolic stress. Herein, the mussel lives in extreme environmental variability, where their ability to cope with perturbations, and build plasticity and adaptive responses, seems based on the genome architecture.

## 4. Conclusions

This study reports the first chromosome-level genome assembly of the native blue mussel *Mytilus chilensis*. This genomic resource was used to identify genome signatures related to the phenotypic plasticity in the mussel population inhabiting contrasting marine environments. Collectively, the putative mutations associated with immune- and metabolism-related genes suggest molecular regulatory mechanisms to reduce the number of genes and their transcriptional activity under stress conditions. This evolutionary strategy can suggest that the expression of those genes has evolved a degree of “frontloading” that potentially pre-adapts the mussel populations to frequent heat and salinity stress, contributing to their physiological tolerance and fitness. We believe that the generated genomic resource must be instrumental for future research on population genomics informing management and sustainable strategies for the Chilean mussel aquaculture.

## Figures and Tables

**Table 1:** Statistics of whole-genome sequence assembly and transcriptome analysis of the blue mussel *Mytilus chilensis* using Illumina, PacBio, and Hi-C.

Types	Method	No. of Reads (Millions)	Read Length	Length (Giga Base Pair)
Genome	PacBio SMRT	63M	20 kb	882 Gbp
	Illumina (Hi-C)	253M	150 bp	37 Gbp
Transcriptome	Illumina (Hemolymph)	38.3M	100 bp	3838 Gbp
	Illumina (Mantle)	37.1M	100 bp	3714 Gbp
	Illumina (Gills)	38.6M	100 bp	3865 Gbp
	Illumina (Digestive gland)	28.2M	100 bp	2823 Gbp

**Table 2:** Genome assembly statistics using HiFi reads and proximity ligation analysis for *Mytilus chilensis*.

Label	Statistics
PacBio assembly	
Assembly size	2,191,715,088
Contig (CTG) N50	206,083
CTGs	13,762
Hi-C mapping	
Total read pairs (RPs) analyzed	253,342,981
High-quality (HQ) * RPs	14.71%
Clustering usable HQ reads per contig (CTGs > 5 kb) *	1215.84
RPs > 10 kb apart (CTGs > 10 kb)	18.68%
Intercontig HQ RPs	44.68%
Same strand HQ RPs	21.50%
Split reads	37.80%

* Number of contigs in scaffolds: 11,868 (100% of all contigs in chromosome clusters, 86.24% of all contigs).

**Table 3:** De novo assembly of the *M. chilensis* genome using proximity ligation (Hi-C).

Chromosome Number	Number of Contigs	Length (bp)
1	988	173,300,526
2	852	140,500,440
3	957	154,573,458
4	871	155,184,769
5	902	143,621,794
6	845	146,028,403
7	946	139,985,977
8	821	134,004,722
9	794	133,130,516
10	821	132,801,123
11	802	131,378,307
12	790	122,972,572
13	735	113,313,251
14	744	117,342,092
Total	11,868	1,938,137,950
N50		134,004,722

**Table 4:** Statistics of the classification results of repeat sequences from the *M. chilensis* genome.

Type	Number of Repeats	Length (bp)	% in Genome
DNA_TE:EnSpm	4175	1,578,431	0.08%
DNA_TE:Harbinger	3592	1,793,590	0.09%
DNA_TE:Helitron	32,469	18,451,639	0.95%
DNA_TE:MuDR	5329	2,668,407	0.14%
DNA_TE:Other	50,021	29,284,120	1.51%
DNA_TE:TcMar	4523	3,828,266	0.20%
DNA_TE:hAT	9902	4,997,238	0.26%
LTR:Copia	3229	1,398,734	0.07%
LTR:Gypsy	9724	12,056,219	0.62%
LTR:Other	8753	12,605,602	0.65%
Low_complexity	38	30,762	0.00%
NonLTR:LINE	124,903	143,701,714	7.41%
NonLTR:SINE	9366	4,939,193	0.25%
Tandem repeat: Satellite and Other	108,470	122,642,591	6.32%
Unknown	767,910	740,296,986	38.17%
All Repeat	1,142,404	1,100,273,492	56.73%

**Table 5:** Basic statistical results of gene structure prediction of relative species.

Species	Number of Genes	Average Transcript Length (bp)	Average CDS Length (bp)	Average Exons Per Gene	Average Exon Length (bp)	Average Intron Length (bp)
*Crassostrea gigas*	63,340	17,784	2008	12.6	3301	286
*Mytilus galloprovincialis*	77,414	10,977	1369	7.8	13,727	260
*Mytilus coruscus*	37,478	14,735	2900	5.9	1290	2727
*Dreissena polymorpha*	68,018	13,316	2603	4.5	1632	1194

**Table 6:** Statistical gene structure prediction for the blue mussel *M. chilensis* genome.

	Type	Number	Average Transcript Length (bp)	Average CDS Length (bp)	Average Exons Per Gene	Average Exon Length (bp)	Average Intron Length (bp)
De novo	Augustus	83,110	10,628	1423	5.74	1423	1941
	SNAP	70,217	36,619	536	9.46	536	4265
	GlimmerHMM	39,700	1973	753	2.85	753	658
	Geneid	49,963	3111	1036	2.79	1036	1160
	Genscan	83,110	10,628	1423	5.74	1423	1941
	GeneMark	81,256	11,018	1301	5.82	1301	270
Homolog	GMAP	250,833	16,107	370	3.10	1649	6891
	spaln	136,264	-	-	7.80	179	-
RNAseq	PASA	18,056	-	-	2.05	504	-
	EVM	63,943	6137	1133	4.54	1133	1414
Final set		34,530	6531	1377	4.92	1377	1316

**Table 7:** Statistics of non-coding RNA annotation for the *M. chilensis* genome.

Type	Copy Number	Average Length (bp)	Total Length (bp)	% of Genome
miRNA	1365	92.8	126,678	0.01%
rRNA	143	118.58	16,957	0.00%
sRNA	275	83.68	23,011	0.00%
snRNA	99	106.71	10,564	0.00%
snoRNA	2267	89.38	202,622	0.01%
tRNA	516	77.44	39,957	0.00%
tRNA pseudogenes	123	72.96	8974	0.00%
All types	4795	89.42	428,763	0.02%

**Table 8:** Statistics of gene function annotation for the *M. chilensis* genome.

	Number	Percentage (%)
Swiss-Prot	24,325	70.45
Nr	25,212	73.01
Nt	3102	8.98
KEGG	22,425	64.94
eggNOG	23,181	80.57
GO	11,606	33.61
Pfam	60,887	96.33
Annotated	27,821	80.57
Unannotated	6709	19.43
Total	34,530	100

## Data Availability

The *Mytilus chilensis* whole-genome sequencing data supporting this study’s findings are available from NCBI under BioProject PRJNA861856. The sequencing data supporting this study’s findings are available in SRA at SRR20966976, SRR20593343, and SRP261955. The benefits from this study accrue from sharing our data and results on public databases as described above. The assembled genome and the genome annotation results were deposited in the Figshare database [93].

## Supplementary Materials

The following supporting information can be downloaded at: https://www.mdpi.com/article/10.3390/genes14040876/s1, Figure S1: Distribution of Simple Sequence Repeats (SSR) identified by SSR Finder in the *M. chilensis* genome; Figure S2: Dot-blot analysis between *M. chilensis* and *M. coruscus* chromosomes, and macro-syntenic relationships; Figure S3: Temporal and spatial variability of Sea Surface Temperature (SST) around Chiloé island, and at sites Yaldad and Cochamo, over the past two decades.
