# Supplementary material for: Chromosome-Level Genome Assembly of the Blue Mussel Mytilus chilensis Reveals Molecular Signatures Facing the Marine Environment
Source: Genes (Basel). 2023 Apr 7;14(4):876. doi: 10.3390/genes14040876 (PMC10137854; doi:10.3390/genes14040876)
Supplement: Supplementary file 1 [file genes-14-00876-s001.zip › genes-2291516-supplementary.pdf]

Supplementary Figures

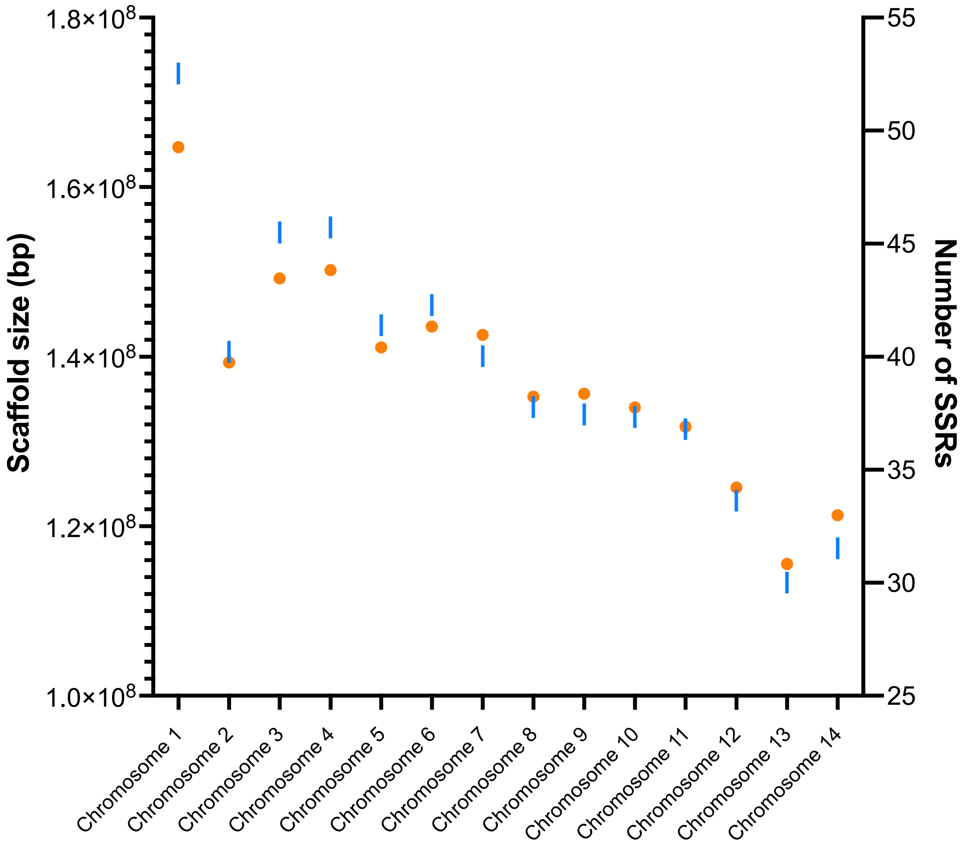

Figure S1. Distribution of Simple Sequence Repeats (SSR) identified by SSR Finder in the *M. chilensis* genome. SSRs were annotated through the 14 pseudo-chromosomes with a total number of 441.494 sequences. Vertical blue lines and orange dots represent the scaffold size (bp) and the number of SSRs, respectively.

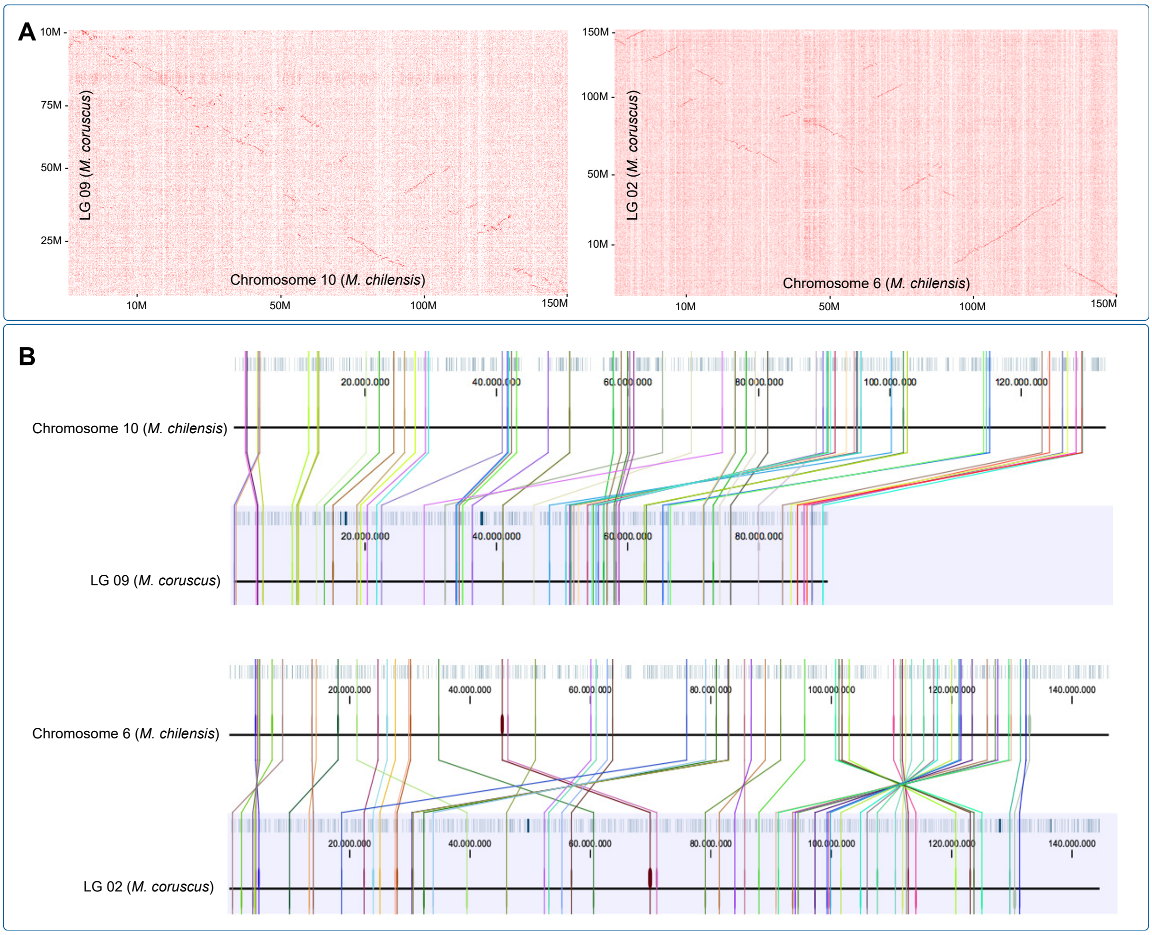

Figure S2. (A) Dot-blot analysis between chromosome 10 vs. LG 09, and chromosome 6 vs. LG 02 for *M. chilensis* and *M. coruscus*, respectively. (B) Macro-syntenic relationships between mussel chromosomes.

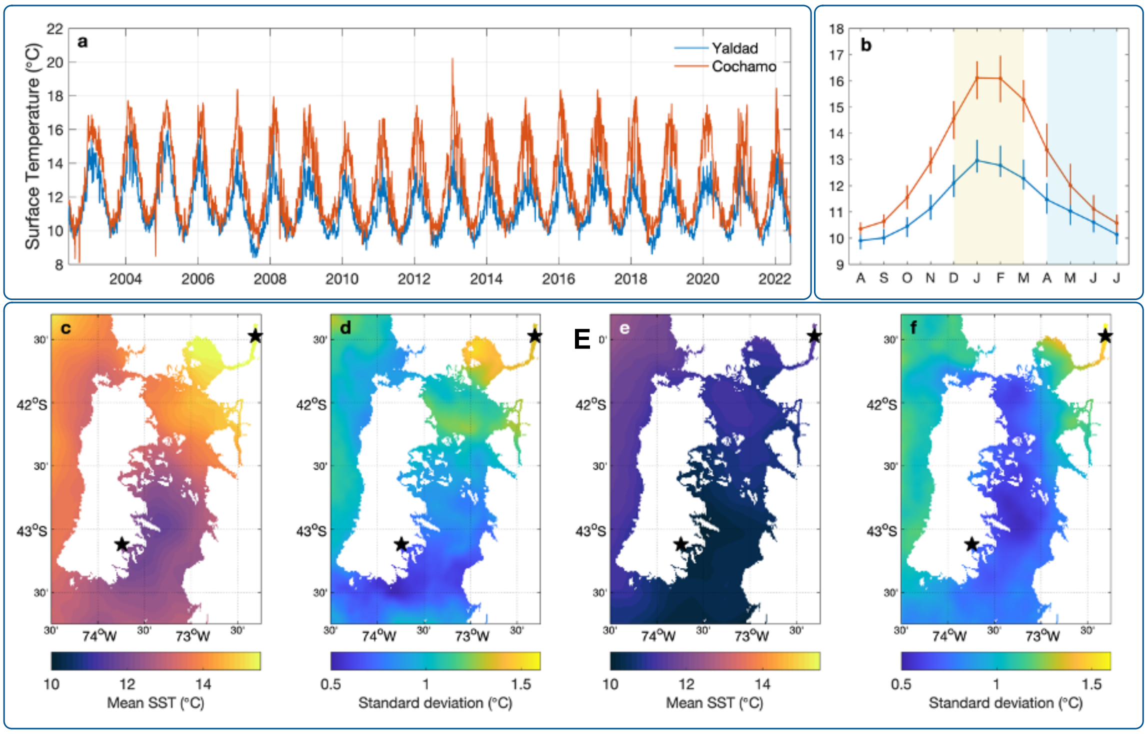

Figure S3. Temporal and spatial variability of Sea Surface Temperature (SST) around Chiloé island, and at sites Yaldad and Cochamo, over the past two decades. (a) Daily time series of SST extracted from satellite-derived data for both sites. (b) Monthly medians computed from data in (a), showing the first and third quartiles as error bars; note that the sequence of months shown on the x axis begins in August and ends in July. Shaded areas in (b) indicate summer (yellow) and fall-winter (blue) periods for which mean fields and the associated standard deviations are shown in (c-f). Maps in (c) and (e) correspond to mean SST computed for December 2017 – March 2018 and 1 April 2018 to 31 July 2018, respectively. Maps in (d) and (f) are the corresponding standard deviations. Stars indicate the location of sampling sites.
